# Online recommenders’ anthropomorphism improves user response to hedonic and benefit-based product appeals through the recommenders’ perceived ability to learn

**DOI:** 10.1371/journal.pone.0287663

**Published:** 2023-06-30

**Authors:** Wojciech Trzebiński, Beata Marciniak, Eliza Kulczycka

**Affiliations:** Department of Market, Marketing and Quality, Collegium of Management and Finance, SGH Warsaw School of Economics, Warsaw, Poland; University of Castilla-La Mancha: Universidad de Castilla-La Mancha, SPAIN

## Abstract

Previous studies reveal the limited effectiveness of benefit-based and hedonic-based product recommendations provided by online recommenders, and recommender anthropomorphism is considered a remedy. This paper aims to investigate the positive effect of anthropomorphism by involving the online recommender’s perceived ability to learn as a mediator. Based on schema congruity theory, perceived benefit/hedonic appeals appropriateness is considered a dependent variable. In Study 1, subtle anthropomorphic cues within an online recommender had a positive effect on perceived benefit-appeals appropriateness through the perceived ability to learn. Study 2 demonstrated the positive relationship between perceived anthropomorphism and perceived hedonic-appeal appropriateness, with the mediating role of the perceived ability to learn. The results advance the knowledge about consumer response to online recommenders from the perspective of anthropomorphism and schema congruity theory. Marketers and consumer organizations are advised on how to deal with online recommender systems providing benefit and hedonic appeals.

## Introduction

Online recommenders are increasingly used in e-commerce websites. The compound aggregated growth rate of the global recommender engine market for the period 2021–2026 was expected at the level of 37.5% [1]. Consumers expect personalized communications from businesses (e.g., 71% of positive answers in the US adult population [2]). However, studies show that machine recommenders vs. human recommenders are distinguished by consumers, which produces certain limitations in the persuasiveness of the former [3–6]. Specifically, product recommendations based on benefits [3, 4] and referring to hedonic purchase motives [5–7] are less effective when provided by machine vs. human recommenders.

When consumers perceive machine recommenders more as humanlike (i.e., higher perceived anthropomorphism), the latter are more persuasive [8], especially in terms of benefit and hedonic appeals [4, 6]. This suggests that anthropomorphizing online recommenders or referring to their anthropomorphic perception is a promising path to improve the effectiveness of e-commerce product communication. Therefore, a better understanding of the mechanisms standing behind this positive effect of anthropomorphism, apart from being theoretically important, may help online marketers in developing effective online product recommenders.

A substantial mechanism contributing to the positive effect of anthropomorphism on consumer reactions to benefit appeals and hedonic appeals of online recommenders may be related to their perceived ability to learn about consumer needs and preferences [9, 10]. The previous related studies [3–6] did not address this possibility. Therefore, the current research aims to investigate the role of the perceived online recommender’s ability to learn in the relationship between a recommender’s anthropomorphism and consumer reaction to the recommender’s benefit and hedonic product appeals.

In the current research, it is proposed that the perceived online recommender’s ability to learn mediates the relationship between perceived recommender anthropomorphism and consumer reaction to online recommenders’ benefit appeals and hedonic appeals [4, 5, 10–12]. Drawing on schema congruity theory [13–16], the current research views consumer restraint to benefit and hedonic appeals of online recommenders as a result of incongruity between the "nonhumanness" schema of machines [15] and the human perspective of using and enjoying products. Consequently, the current research involves perceived online recommender appropriateness as a dependent variable.

The two studies demonstrate that the perceived online recommender’s ability to learn mediates the relationship between perceived recommender anthropomorphism and the perceived appropriateness of benefit and hedonic appeals of the recommender. The current results extend the existing knowledge about the role of anthropomorphism in consumer response to machine recommenders [3–6] by introducing the perceived ability to learn as a mediator. Those results advise marketers on how to improve the effectiveness of online recommenders. At the same time, consumer protection activists and authorities are guided on how to defend consumers against online recommendations which may appear relevant but may not necessarily be so.

## Conceptual background

### AI-based online product recommenders

Recommender systems are applications suggesting relevant items [17] to support user choices from large groups of alternatives [18]. Such product recommenders are widely used within e-commerce websites [19] to solve information overload issues [20]. Recommender systems predict user product preferences based on various data, like online user behavior, product ratings provided by similar users or friends, preferences explicitly stated by users, and contextual data [17, 20–24]. Recommender systems use numerous AI methods [25], enabling them to perform complex tasks. Although consumer choice from a recommended set of alternatives is not free of decision biases [26], AI recommenders are believed to improve the rationality of consumer purchase decisions [22]. In turn, consumer recognition of the quality of provided recommendations may lead consumers to adopt those systems [18, 25, 27].

While, in general, AI-based online product recommenders can support various favorable user reactions, including product purchase and loyalty towards the website [18, 20, 28], this influence appears to be uneven. In certain cases, consumers may react to recommender systems, compared to humans (like salespeople, influencers, or other consumers), less positively. For example, consumers tend to rely less on recommender systems (vs. humans) when products are experience-type [7]. Consumers may also react differently depending on the type of product appeal. Specifically, in the case of machine (vs. human) recommenders, attribute appeals are more persuasive than benefit ones [3, 4], and utilitarian appeals are more persuasive than hedonic ones [5, 6].

### Recommenders and schema incongruity theory

According to schema congruity theory [13, 14, 16], people negatively react when a stimulus is significantly incongruent with the existing and activated schema, and this incongruency remains unresolved. Schema congruity theory can be applied to AI-based systems perception as consumers may use a "nonhumanness" schema of digital agents [15]. Therefore, when consumers recognize a dis-match between the output of the AI-based recommender and the "nonhumanness" schema applied to it, an unresolved incongruency may appear, producing a negative reaction, i.e., perceiving inappropriateness of the product recommendations [4]. Specifically, this may happen when the recommendations provided by machine recommenders do not match the "nonhumanness" schema, e.g., being related to the consumer perspective of using products (benefit appeals) or taking pleasure in them (hedonic appeals). This way, the incongruity perspective can help explain different reactions to machine and human recommenders.

### Perceived recommender anthropomorphism

Apart from distinguishing between AI-based and human recommenders, another mechanism may play a role in consumer reactions. According to Computer as Social Actors (CASA) theory [29], people interact with computers treating them as other people by applying social rules and expectations. Likewise, user-synchronous robot movements were positively related to user attitude toward a robot [30]. Consumers tend to trust AI-based recommenders more when the former disclose their personal information, and the conversation is reciprocal [31] and apply gender stereotypes when evaluating recommender competencies [32]. Treating AI-based recommenders as humans may resolve the incongruity between the recommender output and the "nonhumanness" schema. In such cases, machine recommenders become expectable to play human roles, like referring to the human perspective of product benefits and human-specific states like pleasure. This may improve consumer reactions to benefit-based and hedonic-based recommendations.

The tendency to perceive machines as humanlike [33–35] may strengthen treating AI-based systems like humans, as posited by CASA. Consequently, one may expect that the degree consumers neglect benefit-based or hedonic-based recommendations provided by an AI recommender is diminished by the level of its anthropomorphism [3–6], that is, the degree to which the system is perceived as similar to a human (see [36]). Numerous previous studies demonstrated the positive influence of computer agent anthropomorphism on identification with the agent [33] and its outputs [10], consumer trust [36–39], and purchase intent [40].

Marketers can anthropomorphize recommender systems at various levels. For example, those systems may provide explicit anthropomorphic cues like the form of an avatar [41] and display of social communication style [42], emotions, empathy, or warmth [37, 43–45]. On the other hand, recommender systems may be subtly anthropomorphized using human-to-human conversation conventions like first-person statements [10, 46–48] or apologizing [49]. Eventually, users may anthropomorphize recommender systems driven by a general tendency to perceive inanimate objects as having human traits [50]. From this perspective, even the mere intelligence of a system (e.g., manifested in recommendation quality) may act as an anthropomorphic feature [51].

## Research gaps and hypothesis development

### Benefit product appeals of AI-based recommenders

Consumers may organize their product category knowledge in the form of a continuum from the most concrete information directly related to products (i.e., their attributes), through information on product benefits, to the most abstract information about consumer goals and values [52–55]. More concrete terms are instrumental to the more abstract ones in this knowledge structure. Specifically, product attributes may be interpreted as leading to benefits, and benefits result in achieving goals and values. From that perspective, product benefits are closer to consumer self-knowledge of what is important in consumers’ life and what they need [56].

In line with the general "nonhumanness" schema of machines [15], machines (compared to humans) are typically perceived as not possessing their own goals but designed to serve humans [4]. Likewise, consumers appreciate the preciseness of information provided by AI recommenders [57]. Referring to goals through product benefits may be, thus, perceived as less congruent with a typical machine schema. In line with schema congruity theory [13–16], AI-system (vs. human) product recommendations based on benefit (vs. attribute) appeals may be perceived less positively (or more negatively). This effect was thoroughly examined by Kim & Duhachek [3, 4], who demonstrated that attribute-based product recommendations were more persuasive than benefit-based ones when provided by a machine recommender (e.g., on a website), and no effect of appeal type was visible for a human recommender. Moreover, in line with the above considerations on recommender anthropomorphism, Kim & Duhachek [4] found that the preference for attribute-based system recommendations disappeared when the machine was more humanlike. Specifically, an attribute-based recommendation provided by a service robot without a human name was more persuasive than a benefit-based one, while a benefit-based recommendation provided by a service robot with a human name and described as being "conscious" (a humanlike trait) was more persuasive than an attribute-based one. In line with congruency theory, Kim & Duhachek [4] also demonstrated the mediating role of perceived recommendation appropriateness in the above relationship.

Despite the abundant evidence by Kim & Duhachek [4] on the role of anthropomorphism in consumer reaction to benefit appeals provided by machine recommenders, two meaningful issues call for further investigation. First, Kim & Duhachek’s [4] examination of the anthropomorphism effect did not use an online product recommender but a service robot. Second, and more importantly, it remains uncertain how the effect of recommender anthropomorphism influences the reaction to benefit appeal through perceived recommender ability to learn. Determining this relationship could improve the understanding of the mechanism of consumer preference for benefit-based AI online recommendations and help marketers to design recommenders that are more persuasive using benefit language. To address this gap, the current study examines the downstream effects of online recommender anthropomorphism on the perceived recommender’s ability to learn and the perceived appropriateness of the recommender’s benefit-based recommendations.

According to the model of perceived intelligence by Rijksdijk et al. [9], object intelligence is perceived through several dimensions, one of which is the ability to learn, defined as the degree to which an object is capable of storing information about the environment and adapting to it. In the context of recommender systems, the ability to learn is related, among others, to acquiring information on user product preferences and providing recommendations based on that knowledge [10]. Consumers may conceptualize object intelligence based on how they view human intelligence [11, 12]. If learning about other people is considered a human property, then perceiving a recommender as more humanlike (i.e., higher recommender anthropomorphism) may make people infer that a recommender is able to understand other people.

When consumers perceive a recommender system as more able to learn about their preferences, they may view it as more appropriate to present benefit-based product recommendations. This is because a system that can better understand its users is capable of relating to their goals and establishing a link between them and the recommended products via product benefits. In other words, a machine recommender that is highly able to learn about its users is less likely to activate the schema of a typical "goal-less" machine and—according to congruity theory [13, 14, 16]–is less likely to produce the negative reaction to benefit appeal. Likewise, in a gym’s advertisements, the message "How to exercise?" (possibly more similar to an attribute appeal as it is oriented on how a gym operates, i.e., what its attributes are) was more persuasive than the message "Why to exercise? (possibly more similar to a benefit appeal as it is oriented on why to use a gym, i.e., what its benefits are) only when it was not suggested that the machine recommender (voice assistant) is generally capable of learning [4]. Put together, it is proposed that perceiving an online recommender as humanlike increases its perceived ability to learn about its users, which in turn improves its perceived appropriateness to provide benefit-based product recommendations. Formally,

**H1. The perceived anthropomorphism of an online recommender is positively related to the perceived appropriateness of benefit-based recommendations provided by that recommender.**
**H2. The relationship between perceived anthropomorphism and perceived appropriateness of benefit-based recommendations (H1) is mediated by the perceived recommender ability to learn.**

### Hedonic product appeals of AI-based recommenders

Consumers may be motivated to buy products because of their utility in achieving some further goals (utilitarian motives) or because they give pleasure (hedonic motives) [58, 59]. Hedonic/utilitarian purchase motives are considered an important factor in e-commerce (e.g., [60–62]). As pleasure is a human-specific state, machines may be perceived as incompetent to recommend products through hedonic appeals (i.e., those based on hedonic motivation) [5]. Dealing with pleasure is unlikely to match the view of a "pleasureless" machine which may stem from a general "nonhumanness" schema applied by consumers to AI-based systems (cf. [15]). Thus, according to congruity theory [13–16], one may expect that hedonic appeals of recommender systems produce negative reactions. Indeed, Longoni & Cian [5] demonstrated that consumers were more interested in utilitarian-based (but not hedonic-based) recommendations of a computer algorithm than a human. When products were purchased for hedonic motives, recommendations from humans were more persuasive than those from AI recommender depicted as a robot [6]. Moreover, in line with the above considerations on recommender anthropomorphism, humanizing a recommender (by depicting it with a human vs. robot appearance and name) was positively related to the persuasiveness of its hedonic-based product recommendations [6]. Likewise, a chatbot declaring to be a female (vs. declaring to be a genderless AI) was evaluated as more competent in recommending hedonic products [32].

Despite strong evidence that Wien & Peluso [6] provided for the relationship between recommender anthropomorphism and consumer reaction to hedonic appeals, two meaningful issues call for further investigation. First, Wien & Peluso [6] did not involve the perceived appropriateness of hedonic appeals, so it is not well evidenced whether the recommender’s anthropomorphism reduces incongruity between hedonic appeal and a "pleasureless" machine schema. Second, it remains unknown whether the positive effect of anthropomorphism on consumer reaction to hedonic appeal is caused by the perceived recommender ability to learn about a consumer. Determining the role of perceived appropriateness and ability to learn in the effect of recommender anthropomorphism could improve the understanding of the mechanism of consumer preference for AI-based hedonic-based recommendations and help marketers to design recommenders that are more persuasive in appealing to hedonic motives. To address this gap, the current study examines the downstream effects of online recommender anthropomorphism on the perceived recommender’s ability to learn and the perceived appropriateness of the recommender’s hedonic-based recommendations.

As argued in the case of benefit appeals, a recommender that is more humanlike may be perceived are more able to understand user needs and preferences. As a result, that kind of recommender may be perceived as more able to understand human pleasure. In line with that, perceived recommender competence to recommend a product mediated the relationship between hedonic vs. utilitarian goal activation and consumer interest in the recommendations [5]. Therefore, it is proposed that the online recommender’s perceived anthropomorphism increases its perceived ability to learn about its users, which in turn improves its perceived appropriateness to provide hedonic-based product recommendations. Formally,

**H3. The perceived anthropomorphism of an online recommender is positively related to the perceived appropriateness of hedonic-based recommendations provided by that recommender.**
**H4. The relationship between perceived anthropomorphism and perceived appropriateness of hedonic-based recommendations (H3) is mediated by the perceived recommender ability to learn.**

**Fig 1:**
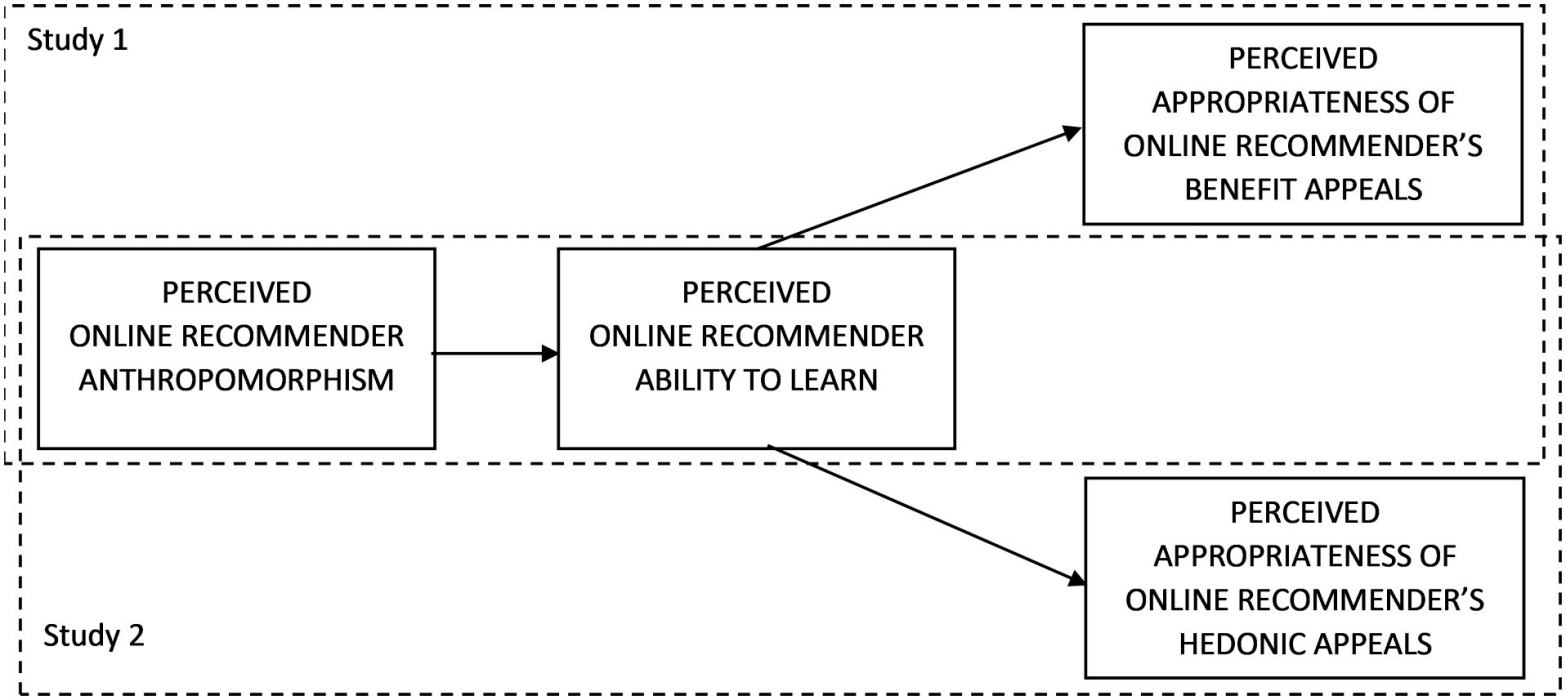
Conceptual framework.

The presented conceptual model (Fig 1) is examined in two studies involving perceived recommender anthropomorphism, ability to learn, and appropriateness of the product recommendations. Study 1 pertains to benefit-based recommendations and tests H1-H2, while Study 2 deals with hedonic-based recommendations to test H3-H4. Both studies used smartphones as a product category as they are highly engaging [63–65] and related to both hedonic and utilitarian purchase motives [6]. Young adults were a studied population in both studies as this consumer group extensively uses smartphones [66].

## Methods and results

### Study 1—The online recommender anthropomorphism and the perceived appropriateness of benefit product appeals

#### Participants

Two hundred fifty young adults (60.4% women, aged between 20 and 30, M = 26.1, SD = 2.9; 66.8% aged at least 25) using smartphones on a daily basis, and having purchased online in the last three months, participated in an online study (see Table 1 for sample characteristics). To increase sample homogeneity for investigating general relationships between constructs (cf. [67]), having at least a high school education (high school: 18.4%, post-secondary: 7.4%, university: 74.0%), and working or studying (72.8% declared they worked; 26.0% declared they studied) were applied as the additional recruitment criteria. The participants were recruited via an online research panel (ePanel.pl) and were Polish native speakers. The study was conducted in Polish. The data collection was part of a larger study on consumer response to AI-based recommendations; the other part, related to trust in recommendations, was reported by Trzebiński & Marciniak ([10], Study 2).

**Table 1:** Sample characteristics (Study 1 and Study 2).

	Study 1		Study 2	
	Frequency	Percent	Frequency	Percent
**Gender**				
**Females**	151	60.4	87	85.3
**Males**	99	39.6	15	14.7
**Age**				
**<25**	83	33.2	60	58.8
**≥25**	167	66.8	42	41.2
**Occupation**				
**Working**	182	72.8	50	49.0
**Studying**	65	26.0	89	87.3

#### Ethics statement

The procedure was carried out under applicable guidelines and regulations and approved by the SGH Warsaw School of Economics Ethics Committee (approval no. 15_2022). Appropriate informed written consent has been collected from the participants via the survey form.

#### Procedure

The participants were asked to test the fictitious online recommender system. They were instructed that the system is a prototype of a new platform called "Electroselect," which would recommend smartphones [10]. The participants were randomly assigned between the four versions of the platform, according to the 2(recommender anthropomorphism: high vs. low) × 2(website type: e-commerce vs. consumer organization) design (see S1 Table for the demographics across the conditions). In the high-anthropomorphism condition (*N* = 121), the recommender displayed messages in the first person (e.g., "I have prepared a recommendation."), while in the low-anthropomorphism condition (*N* = 129), there were no first-person messages (e.g., "A recommendation has been prepared.") [10]. A similar approach was applied by Adam et al. [46], Aggarwal & McGill [47], and Wan et al. [48]. Additionally, in the high-anthropomorphism condition, a head contour was displayed by the recommender’s messages. However, there was no face outline to avoid eliciting emotions and associations with a certain facial appearance [10]. In the e-commerce condition (*N* = 123), the recommender was described as being an online shop selling consumer electronics. In contrast, in the consumer organization condition (*N* = 127), the recommender was described as a platform run by an independent organization supporting consumers in buying consumer electronics products [10]. This manipulation was used to explore how website type may influence the studied effects. Representative screenshots of the product recommendation platform for all experimental conditions are shown in S1 Fig.

At the beginning of the survey, the recommender asked the participants to help improve its recommendations by providing information about the values that are important to them [10]. As that ability-to-learn cue may be perceived as congruent with anthropomorphic cues [10], it was intended to enhance the effect of the latter. Then, the participants were presented with product recommendations in the form of six smartphone descriptions presented in two sets. The presented models were the same for all participants [10]. This way, the effect of anthropomorphism was isolated from the effect of the actual recommendation quality that might disturb the studied relationships (cf. [27]). A similar approach was applied in the previous related studies by Kim & Duhachek [3, 4], Longoni & Cian [5], and Wien & Peluso [6]. The first set of smartphone descriptions contained three ones related to smartphone attributes (i.e., reliability, fastness, and picture quality). In contrast, the second set contained the other three descriptions, which were related to the corresponding smartphone benefits (i.e., secured web access, the possibility to use the newest applications, and entertainment, respectively). The attributes were designed to be instrumental to the corresponding benefits (e.g., a smartphone’s reliability may help secure web access) to reduce the possible disturbance of the effect of benefit vs. attribute appeals caused by the difference in the goals associated with the presented attributes and benefits.

After reading the descriptions of the recommended smartphones, the participants filled out a questionnaire. They rated the appropriateness of the recommender to present each smartphone description, recommender anthropomorphism, and ability to learn. The dependent variable (appropriateness) was measured first to avoid self-generated validity issues [68, 69]. After measuring the studied variables, the participants reported the realism of the situation of using the recommender system ("Could a similar situation in which a website recommends products for you happen in your real life?"; 80.0% of the participants confirmed that "very similar" or "similar" situation could happen) and easiness to imagine that situation ("Was it easy to imagine yourself in the situation of evaluating smartphones recommended by a website?"; 80.0% of the participants marked "very easy" or "easy"). The questionnaire ended with demographics.

#### Measurements

Perceived online recommender appropriateness to recommend product descriptions was measured for the benefits and attributes separately. For each smartphone description presented by the recommender, the participants rated the appropriateness ("Does Electroselect have the appropriate knowledge about you and your preferences to encourage you to consider the recommended smartphones by creating the following descriptions, especially for you?") with a slider scale coded from 0 ("does not have the appropriate knowledge") to 100 ("has the appropriate knowledge"). Similarly, an item containing the word "appropriate" was used in the measurement scale for perceived appropriateness by Roy & Cornwell [70]. In the current study, a single-item measurement was applied as the participants had to rate each of the six smartphone descriptions, and a multi-item measurement might entail too much fatigue. Given the large number of measurements in the study, we intended to keep the questionnaire as short as possible to improve the quality of the collected data. A similar approach was applied by Drossos et al. [71]. Although single-item measurements do not allow for reliability assessment, they are considered acceptable if the measurement is "double-concrete" [72]—that is, the attribute and the object are concretely defined. In our case, the attribute, represented by the question about appropriateness, was precisely formulated (as presented above), and the explicitly presented smartphone descriptions were the objects. As each description pertained to a different aspect of a smartphone, we did not consider their appropriateness ratings as constituting a measurement scale. Instead, each rating was a separate measurement that pertained to a particular object (i.e., a smartphone description) representing a given aspect of a smartphone. Those measurements were grouped into indexes, similar to Han et al. [73]. Specifically, the measurements for benefit-based descriptions were averaged into a single index of perceived appropriateness of benefit appeals. The corresponding attribute-based descriptions were averaged into a single index of perceived appropriateness of attribute appeals.

Perceived online recommender ability to learn was measured with four items based on Rijsdijk et al. [9]. The ratings were expressed on a slider scale coded from 0 ("totally disagree") to 100 ("totally agree") [10]. Perceived online recommender anthropomorphism was measured with nine items based on Qiu & Benbasat [39], Rijsdijk et al. [9], and Wan et al. [48], using the same response format as above [10].

#### Data analysis

To assess the measurement scales (i.e., perceived recommender ability to learn and perceived online recommender anthropomorphism), we used Exploratory Factor Analysis (EFA; assumptions checked with Kaiser-Meyer-Olkin (KMO) test of sampling adequacy and Bartlett’s sphericity test), Confirmatory Factor Analysis (CFA; measurement scales items normality was assessed using standard acceptable levels for skewness (±2) and kurtosis (±2) [74]), and Cronbach’s Alpha. The analyses for manipulation checks and hypothesis testing included standard demographic covariates (gender and age) to avoid potential disturbances caused by differences across experimental conditions. We repeated those analyses without the covariates. ANOVA/ANCOVA was used for manipulation check (experimental conditions as factors and perceived online recommender anthropomorphism as a dependent variable). To test H1, we used ANOVA/ANCOVA (experimental conditions as factors and perceived recommender appropriateness of benefit appeals as a dependent variable) and repeated measures ANOVA/ANCOVA (experimental conditions as between-subject factors, appeal type (benefit vs. attribute) as a within-subject factor, and perceived recommender appropriateness as a dependent variable). Equality of variance was checked with Levene’s test (for ANOVA/ANCOVA) and Box’s test (for repeated measures ANOVA/ANCOVA). Dependent variables normality was tested for each cell with skewness (±2) and kurtosis (±2). We tested H2 based on a serial mediation model using Structural Equation Modelling (SEM); recommender anthropomorphism condition as an independent variable, gender, age, and website type as covariates, perceived recommender anthropomorphism as the first-stage mediator, perceived recommender ability to learn as the second-stage mediator, and perceived appropriateness of benefit appeals as a dependent variable), as it is suitable for including latent variables [75]. In our analysis, latent variables are used to represent the mediators. Except for CFA and SEM, all the analyses were performed using IBM SPSS Statistics 28.0.1.0(142) software. We used STATA 17.0 for CFA and SEM.

#### Results

*Measurement scales assessment*. The perceived online recommender ability to learn and perceived online recommender anthropomorphism measurement scales were assessed with Confirmatory Factor Analysis (CFA). All items had acceptable skewness (ranging from -.946 to .441) and kurtosis (ranging from -1.206 to .607). After modifying the model based on modification indices to reach the acceptable fit, we dropped one item from the measurement scale for perceived online recommender ability to learn ("I think that based on the information collected from me, Electroselect was able to adapt to me and my preferences.”) The fit statistics of the final model were: χ^2^(48) = 119.055, p < .001, χ^2^/df = 2.5, RMSEA = 0.08, CFI = 0.98, TLI = 0.98, SRMR = 0.03. The latent variables reached acceptable Average Variance Extracted (AVE) (AVE_ability to learn_ = .769, AVE_anthropomorphism_ = .831). Moreover, both AVE square roots for the perceived ability to learn and perceived anthropomorphism (which equaled .877 and .912, respectively) were above the correlation between the latent variables (r = .511), indicating discriminant validity. The measurement scales showed satisfactory reliability as determined with Cronbach’s Alpha (α) and Composite Reliability (CR) (for the perceived ability to learn: α = .909, CR = .909; for the perceived anthropomorphism: α = .978, CR = .978). See the details in Table 2.

**Table 2:** Measurement scales (Study 1).

Variable	Measurement item	CFA factor loadings	Cronbach’s Alpha	CR	AVE
Perceived online recommender ability to learn	Electroselect collected information from me to determine my preferences.	.846	.909	.909	.769
	Electroselect used interaction with me to learn about my preferences.	.891			
	Electroselect collected information from me to learn something about my needs.	.894			
Perceived online recommender anthropomorphism	An interaction with Electroselect looks like an interaction with a human being.	.843	.978	.978	.831
	Electroselect functions as a human being.	.885			
	While using Electroselect, I had an impression I interacted with a human being.	.913			
	While using Electroselect, I had an impression that Electroselect had a personality.	.919			
	While using Electroselect, I felt human warmth.	.938			
	While using Electroselect, I had an impression that Electroselect is sociable to some degree.	.889			
	While using Electroselect, I experienced a bit of human tenderness.	.944			
	While interacting with Electroselect, I perceived Electroselect as almost a human being.	.949			
	While interacting with Electroselect, I thought about Electroselect a bit like it was a human being.	.919			

*Manipulation check*. A two-way ANOVA, with the manipulated variables as factors, gender and age as covariates, and perceived online recommender anthropomorphism as a dependent variable, was run to check the anthropomorphism manipulation. In line with the expectations, the perceived anthropomorphism was higher in the high-anthropomorphism (vs. low-anthropomorphism) condition (M_high_ = 45.703, SD = 28.386, M_low_ = 33.629, SD = 25.036, F(1,144) = 12.616, *p* < .001; Levene’s test of equality of error variances: *p* > .06; partial eta squared = .049; skewness ranged from -.062 to .388, kurtosis ranged from -1.208 to -.823). The effect of the anthropomorphism manipulation remained significant after dropping the covariates (F(1,146) = 12.814, *p* < .001; Levene’s test of equality of error variances: *p* > .05; partial eta squared = .050). No effect of website type occurred (*p*’s> .7).

**Fig 2:**
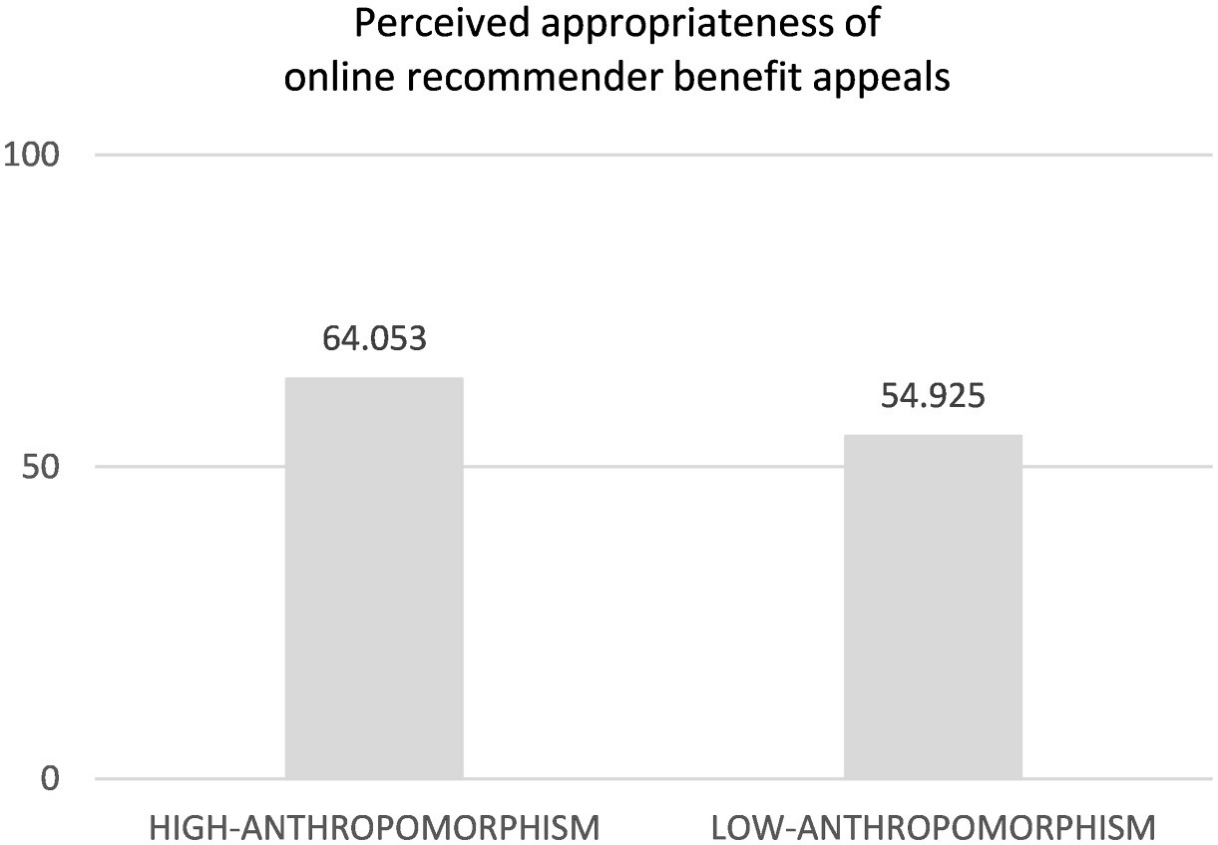
Perceived appropriateness of online recommender benefit appeals and recommender anthropomorphism (ANOVA, Study 1).

*Hypothesis testing*. To test H1 (predicting the positive relationship between the perceived online recommender anthropomorphism and perceived appropriateness of its benefit appeals), a two-way ANOVA, with the manipulated variables as factors, gender and age as covariates, and perceived recommender appropriateness of benefit appeals as a dependent variable was conducted. In line with H1, the effect of recommender anthropomorphism was positive (Fig 2, M_high_ = 64.053, SD = 20.160, M_low_ = 54.925, SD = 20.792, F(1,144) = 12.387, *p* < .001, partial eta squared = .048; Levene’s test of equality of error variances: *p* > .8; skewness ranged from -.840 to -.040, kurtosis ranged from -.300 to 1.249). The above effect remained significant after dropping the covariates (F(1,146) = 11.765, *p* < .001; Levene’s test of equality of error variances: *p* > .8; partial eta squared = .046). No effects of website type on perceived appropriateness occurred (*p*’s > .2). No effects of website type on perceived appropriateness occurred (*p*’s > .2).

To rule out the possibility that recommender anthropomorphism equally increased the perceived appropriateness for the attribute- and benefit-based recommendations, a repeated measures analysis, with the manipulated variables as between-subject factors, appeal type (benefit vs. attribute) as a within-subject factor, gender and age as covariates, and perceived recommender appropriateness as a dependent variable, was conducted (perceived recommender appropriateness for attribute-based appeal: skewness ranged from -.595 to -.486, kurtosis ranged from -.201 to .491). The appeal type × anthropomorphism interaction effect was significant (F(1,244) = 4.166, *p* < .042; partial eta squared = .017; Box’s test of covariance of equality matrices: *p* > .4). Crucially, the difference in perceived recommender appropriateness of the benefit appeals between anthropomorphism conditions (high vs. low) was higher than for the attribute appeals (d_benefit appeals_ = 9.128, d_attribute appeals_ = 3.983). The effect of the appeal type × anthropomorphism interaction remained significant after dropping the covariates (F(1,146) = 4.511, *p* = .035; Box’s test of covariance of equality matrices: *p* > .4; partial eta squared = .018). Those results support H1. No effects of website type occurred (*p*’s > .5).

**Fig 3:**
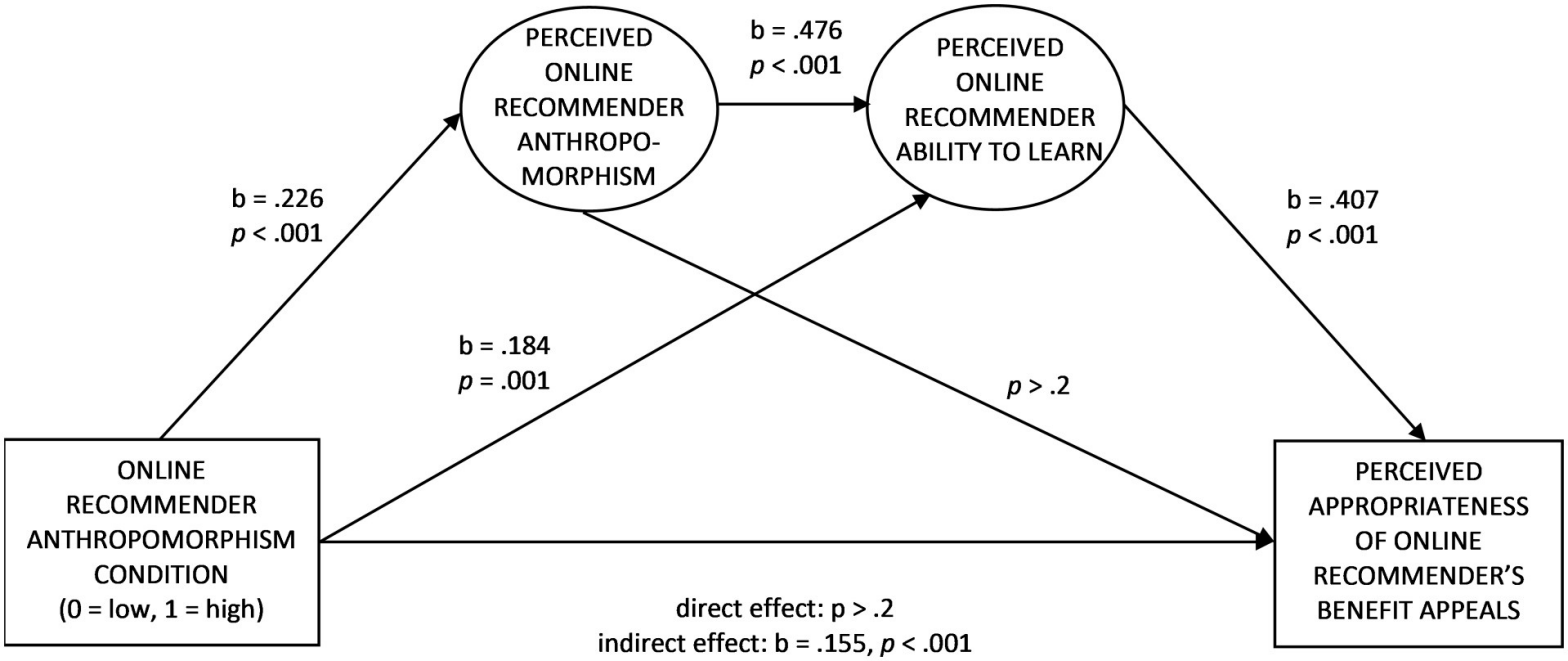
Serial mediation between the online recommender anthropomorphism cues and the perceived appropriateness of its benefit appeals through perceived recommender anthropomorphism and the ability to learn (SEM, Study 1; gender (coded as 0 = man, 1 = woman), age, and website type (coded as 0 = e-commerce, 1 = consumer organization) were covariates; the mediators were included as latent variables).

H2 (predicting the mediation between the perceived online recommender anthropomorphism and the perceived appropriateness of its benefit appeals through perceived recommender ability to learn) was tested with a serial mediation analysis (Fig 3, Structural Equation Modelling (SEM)). The recommender anthropomorphism condition (coded as 0 = low anthropomorphism, 1 = high anthropomorphism) was an independent variable, gender (coded as 0 = man, 1 = woman), age, and website type (coded as 0 = e-commerce, 1 = consumer organization) were covariates, perceived recommender anthropomorphism was the first-stage mediator, perceived recommender ability to learn was the second-stage mediator, and perceived appropriateness of benefit appeals was a dependent variable. The mediators were included as latent variables. The model showed an acceptable fit (χ^2^(103) = 351.924, *p* < .001, χ^2^/df = 3.4, RMSEA = .099, CFI = .94, TLI = .92, SRMR = .03). The anthropomorphism condition had a positive total effect on the perceived appropriateness (b = .219, z = 3.57, *p* < .001) and a positive effect on perceived recommender anthropomorphism (b = .226, z = 3.72, *p* < .001). Perceived anthropomorphism, in turn, had a positive effect on the perceived recommender ability to learn (b = .476, z = 9.12, *p* < .001), and perceived ability to lean had a positive effect on the perceived appropriateness of benefit appeals (b = .407, z = 6.13, *p* < .001). The indirect effect of the anthropomorphism condition on the perceived appropriateness was also positive (b = .155, z = 4.25, *p* < .001). Moreover, the direct effect of the recommender anthropomorphism condition on the perceived appropriateness was non-significant (*p* > .2), indicating full mediation. See details in S2 Table. After dropping the demographic covariates (gender and age), all the subsequent effects in the hypothesized causal chain remained significant (the total effect of anthropomorphism condition on perceived appropriateness of benefit appeals: z = 3.47, *p* < .001; the anthropomorphism condition on perceived recommender anthropomorphism: z = 3.76, *p* < .001; perceived anthropomorphism on perceived recommender ability to learn: z = 8.97, *p* < .001, perceived ability to lean had on the perceived appropriateness of benefit appeals: z = 6.32, *p* < .001, the indirect effect of anthropomorphism condition on perceived appropriateness of benefit appeals: z = 4.18, *p* < .001) while the direct effect of the recommender anthropomorphism condition on the perceived appropriateness was still non-significant (*p* > .2). However, the goodness-of-fit indicators worsened (χ^2^(83) = 322.790, *p* < .001, χ^2^/df = 3.9, RMSEA = .11, CFI = .94, TLI = .92, SRMR = .03), indicating that demographic variables contributed to the studied relationships. Overall, the results support H2.

#### Discussion

Study 1, using subtle anthropomorphism cues as an independent variable and a direct measurement of the perceived appropriateness of benefit appeals as a dependent variable, experimentally demonstrates the positive effect of the perceived online recommender anthropomorphism on consumer reactions to its benefit product appeals. Crucially, the results suggest the mediating role of the perceived online recommender ability to learn in the above relationship.

### Study 2—The online recommender anthropomorphism and the perceived appropriateness of hedonic product appeals

#### Participants

Like the previous study, Study 2 used homogeneous convenience sampling suitable to investigate general relationships between constructs (cf. [67]). One hundred two young adults (85.3% women, aged between 20 and 35, M = 24.8, SD = 2.9; 41.2% aged at least 25) participated in an online study (see Table 1 for sample characteristics). A vast majority of participants used smartphones on a daily basis (99.0%) and had purchased online in the last three months (92.2%). The working (49.0% of the sample) or studying (87.3% of the sample) occupation status was applied as an additional recruitment criterion to increase sample homogeneity. The participants were recruited via social media research groups and were Polish native speakers. The study was conducted in Polish.

#### Ethics statement

The procedure was carried out under applicable guidelines and regulations and approved by the SGH Warsaw School of Economics Ethics Committee (approval no. 15_2022). Appropriate informed written consent has been collected from the participants via the survey form.

#### Procedure

Like in Study 1, the participants were asked to test the fictitious online recommender system, ostensibly a prototype of a new platform, "Electroselect," recommending smartphones. The participants were randomly assigned between the two versions of the platform, according to the one-factor design (recommender anthropomorphism: high vs. low; see S3 Table for the demographics across the conditions). The setup of the high-anthropomorphism condition (*N* = 51) and the low-anthropomorphism condition (*N* = 50) was similar to Study 1 (see S2 Fig for representative screenshots of the product recommendation platform). In both conditions, the recommender was described as being an online shop selling consumer electronics. As in Study 1, the recommender first asked the participants to provide information about their needs. Then, the participants read four smartphone descriptions intended to relate to hedonic motives ("This smartphone will give you the pleasure of …viewing pictures in the best quality, e.g., when you want to enjoy some movie or photos/…using apps based on the newest software, e.g., when you want to enjoy games, music, or video/…making high-quality photos, e.g., when you want to enjoy your memories from a trip or meeting friends/…working fast, e.g., when you want to enjoy quickly-loading games or movies.”). Like in Study 1, the presented models were the same for all participants.

After reading the descriptions of the recommended smartphones, the participants filled out a questionnaire. They rated the appropriateness of the recommender to present each smartphone description, the recommender ability to learn, and anthropomorphism. Like in Study 1, the dependent variable (appropriateness) was measured first. However, the measurement order for two other variables of interest (perceived ability to learn and anthropomorphism) was reversed, as there is an ongoing dispute on the order of measuring mediators (cf. [76]). Then, the participants rated the degree to which the presented smartphone recommendations were hedonic (vs. utilitarian). Like in Study 1, participants rated the realism of the situation of using the recommender system (75.5% of the participants confirmed that a "very similar" or "similar" situation could happen) and the easiness of imagining that situation (79.4% of the participants marked "very easy" or "easy"). The questionnaire ended with demographics.

#### Measurements

Perceived online recommender appropriateness to recommend product descriptions was measured like in Study 1 for each smartphone description presented by the recommender. As the pretest participants indicated it would be easier for them to choose between fewer response options, a five-point response scale was used (1 = "does not have the appropriate knowledge," 5 = "has the appropriate knowledge"). Similar to Study 1, the measurements were averaged into a single index of perceived appropriateness of hedonic appeals.

Perceived online recommender anthropomorphism was measured with the same statements as in Study 1. After the pretest, statements to measure perceived online recommender ability to learn were slightly modified (e.g., "I think Electroselect was able to learn well about me and my preferences" instead of "Electroselect collected information from me to determine my preferences") to improve communication. Similar to the perceived appropriateness measurement, perceived anthropomorphism and ability to learn measurement used shortened response scales (from 1 = "totally disagree" to 7 = "totally agree"). See Table 3 for measurement scale details.

**Table 3:** Measurement scales (Study 2).

Variable	Measurement item	CFA loadings	Cronbach’s Alpha	CR	AVE
Perceived online recommender ability to learn	Electroselect used interaction with me to learn about my preferences.	.706	.871	.878	.708
	After each interaction with me, Electroselect would know me better and better.	.903			
	After each interaction with me, Electroselect would adapt to me and my preferences better and better.	.899			
Perceived online recommender anthropomorphism	An interaction with Electroselect looks like an interaction with a human being.	.841	.961	.961	.733
	Electroselect functions as a human being.	.816			
	While using Electroselect, I had an impression I interacted with a human being.	.901			
	While using Electroselect, I had an impression that Electroselect had a personality.	.912			
	While using Electroselect, I felt human warmth.	.849			
	While using Electroselect, I had an impression that Electroselect is sociable to some degree.	.808			
	While using Electroselect, I experienced a bit of human tenderness.	.898			
	While interacting with Electroselect, I perceived Electroselect as almost a human being.	.865			
	While interacting with Electroselect, I thought about Electroselect a bit like it was a human being.	.807			

Perceived recommendation hedonic (vs. utilitarian) motive was measured separately for each smartphone description with a five-point item (1 = "this description refers more to fun and entertainment provided by the smartphone" to 5 = "this description refers more to usual activities in which the smartphone can help"). The coding was reversed such that the higher values indicate a more hedonic (vs. utilitarian) motive perceived by a participant. A similar measurement was applied by Chen et al. [77] and Wien & Peluso [6]; however, in our items, hedonic and utilitarian motives were referred to together. This was intended to reduce the fatigue of the participants, who had to evaluate each description separately. The measurements were averaged across all smartphone descriptions into a single index.

#### Data analysis

To assess the measurement scales (i.e., perceived recommender ability to learn and perceived online recommender anthropomorphism), we used Exploratory Factor Analysis (EFA; assumptions checked with Kaiser-Meyer-Olkin (KMO) test of sampling adequacy and Bartlett’s sphericity test), Confirmatory Factor Analysis (CFA; measurement scales items normality was assessed with skewness and kurtosis, like in Study 1), and Cronbach’s Alpha. The same variables as in Study 1 (i.e., gender and age) served as covariates in manipulation check and hypothesis testing, and the analyses were repeated without those covariates. ANOVA/ANCOVA was used for the manipulation check (experimental condition as a factor and perceived online recommender anthropomorphism as a dependent variable). Equality of variance was checked with Levene’s test, and dependent variables normality was assessed for each cell with skewness and kurtosis, like in Study 1. We tested H3 and based on mediation and moderated mediation models using ordinary least squares (OLS) regressions in PROCESS macro (PROCESS, [78], Models 4 and 14, 5000 bootstrap samples; perceived recommender anthropomorphism as an independent variable, gender, age, and anthropomorphism condition as covariates, the perceived recommender ability to learn as a mediator, perceived appropriateness of hedonic appeals as a dependent variable, and–only in Model 14—perceived recommendation hedonic (vs. utilitarian) motive as a second-stage moderator), as it is suitable for including continuous moderators and allows analyzing conditional effects with the index of moderated mediation [78, 79]. In our analysis, a continuous moderator is used in Hayes Model 14 [78] to compare the role of hedonic vs. utilitarian motives. Multicollinearity, normality of residuals, and homoscedasticity were tested as in Study 1. The statistical software used was the same as in Study 1.

#### Results

*Measurement scales assessment*. The measurement scales for perceived online recommender ability to learn and perceived online recommender anthropomorphism were assessed with Confirmatory Factor Analysis (CFA). All items had acceptable skewness (ranging from -.306 to 1.021) and kurtosis (ranging from -1.021 to .121). After modifying the model based on modification indices to reach the acceptable fit, we dropped one item from the measurement scale for perceived online recommender ability to learn ("I think Electroselect was able to learn well about me and my preferences.”) The fit statistics of the final model were: χ^2^(48) = 77.167, p < .001, χ^2^/df = 1.6, RMSEA = 0.08, CFI = 0.98, TLI = 0.97, SRMR = 0.06. The latent variables reached acceptable Average Variance Extracted (AVE) (AVE_ability to learn_ = .708, AVE_anthropomorphism_ = .733). Moreover, both AVE square roots for the perceived ability to learn and perceived anthropomorphism (which equaled .841 and .856, respectively) were above the correlation between the latent variables (r = .263), indicating discriminant validity. The measurement scales showed satisfactory reliability as determined with Cronbach’s Alpha (α) and Composite Reliability (CR) (for the perceived ability to learn: α = .871, CR = .878; for the perceived anthropomorphism: α = .961, CR = .961). See details in Table 2

*Manipulation check*. A one-way ANOVA, with the manipulated variable (i.e., recommender anthropomorphism) as a factor, gender (coded as 0 = man, 1 = woman) and age as covariates, and perceived online recommender anthropomorphism (skewness ranged from .504 to .776, kurtosis ranged from -1.158 to .456) as a dependent variable, was run to check the anthropomorphism manipulation. As perceived online recommender anthropomorphism was indifferent to the anthropomorphism conditions (*p* > .7; Levene’s test of the equality of error variances: p > .2; the effect on perceived anthropomorphism remained non-significant after dropping the covariates), all the analyses used perceived anthropomorphism as an independent variable.

**Fig 4:**
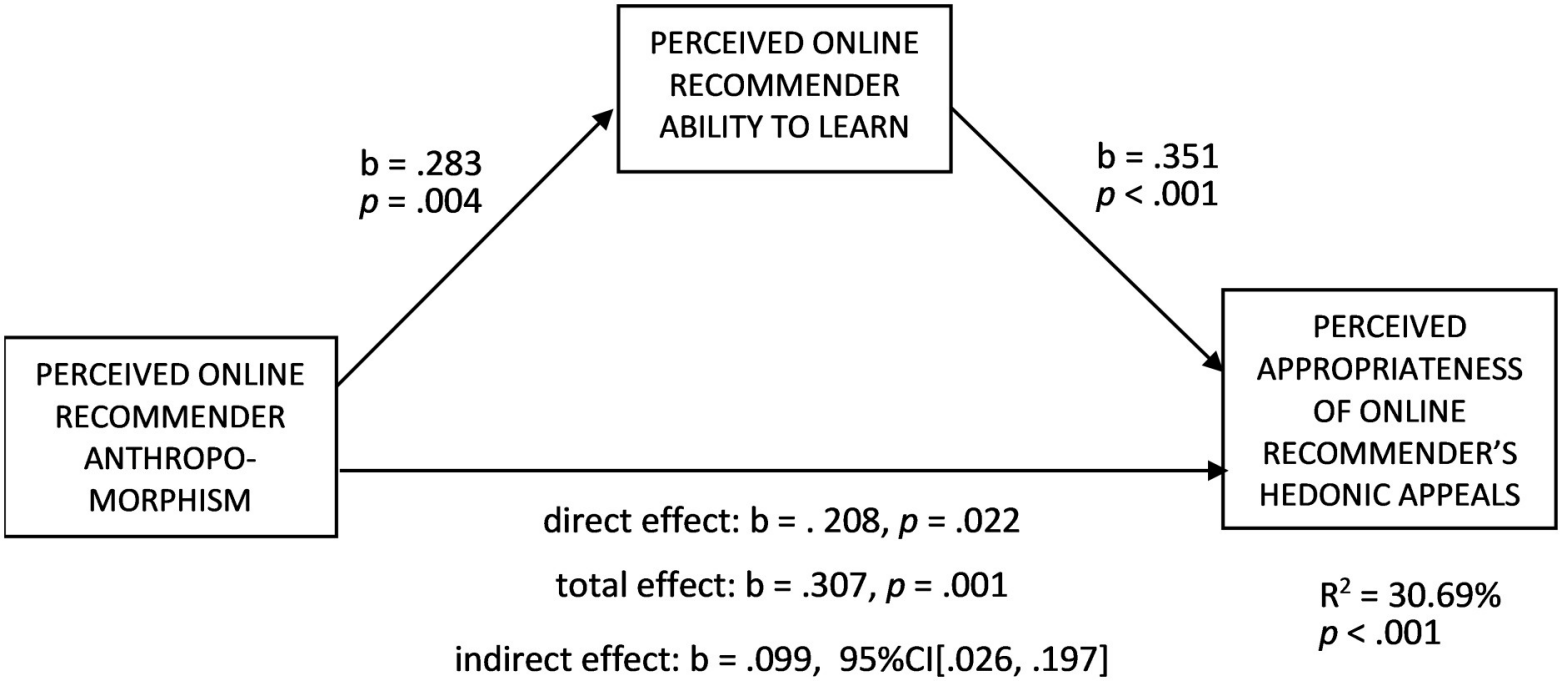
A mediation analysis of the relationship between the perceived online recommender anthropomorphism and perceived appropriateness of its hedonic appeals (perceived recommender ability to learn as a mediator; gender (coded as 0 = man, 1 = woman), age, and anthropomorphism condition (coded as 0 = low, 1 = high) were covariates) (Study 2).

**Fig 5:**
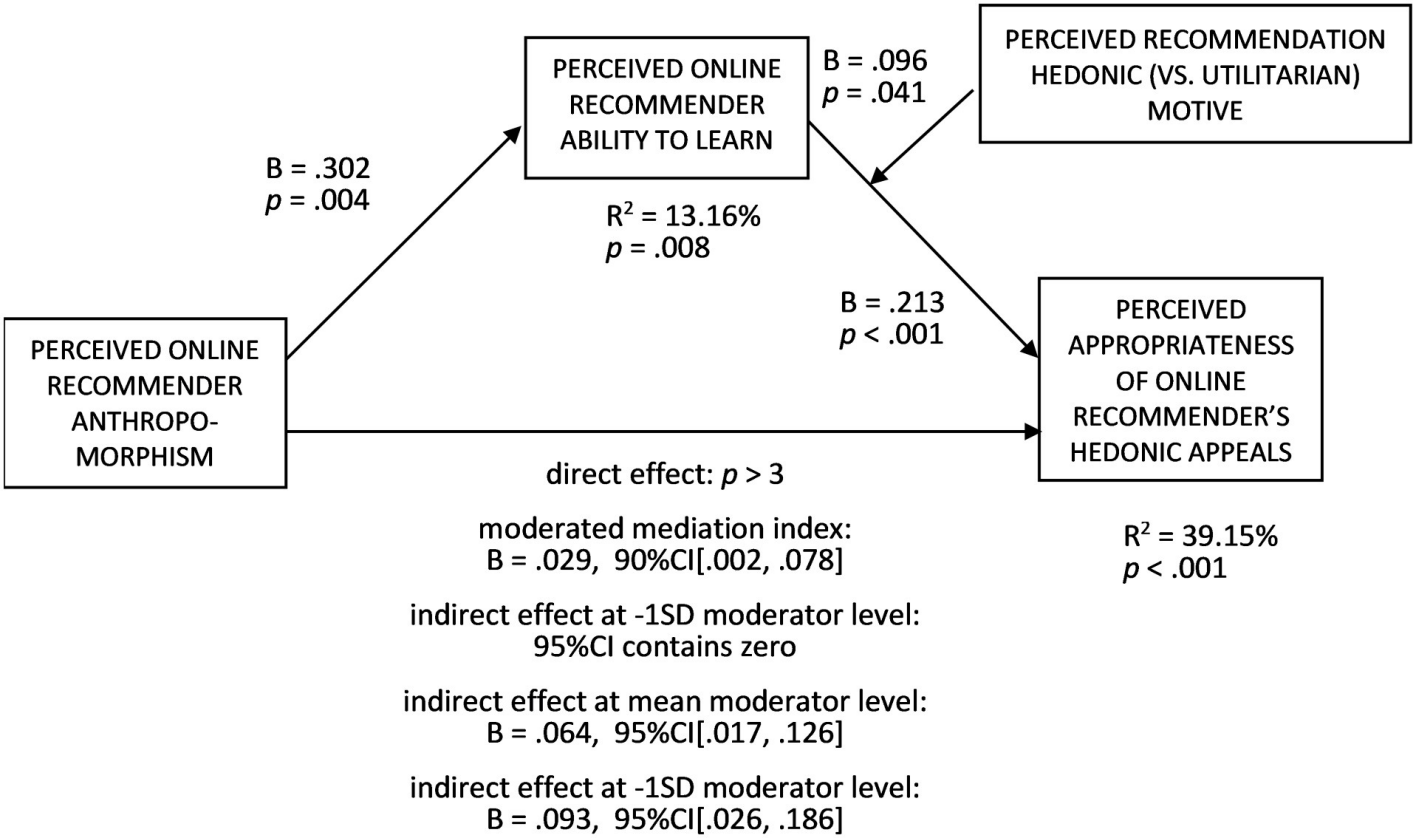
A second-stage moderated mediation analysis of the relationship between the perceived online recommender anthropomorphism and perceived appropriateness of its hedonic appeals (perceived recommender ability to learn as a mediator, perceived recommendation hedonic (vs. utilitarian) motive as a second-stage moderator; gender (coded as 0 = man, 1 = woman), age, and anthropomorphism condition (coded as 0 = low, 1 = high) were covariates) (Study 2).

*Hypothesis testing*. To test H3 (the positive relationship between the perceived online recommender anthropomorphism and perceived appropriateness of its hedonic appeals) and H4 (predicting the mediating role of perceived online recommender ability to learn in the above relationship), we conducted a mediation analysis (Fig 4, PROCESS, Model 4, [78], 5000 bootstrap samples). The perceived recommender anthropomorphism was an independent variable, gender, age, and anthropomorphism condition (coded as 0 = low, 1 = high) were covariates, the perceived recommender ability to learn was a mediator, and perceived appropriateness of hedonic appeals was a dependent variable (VIFs < 1.3). The perceived anthropomorphism had a positive total effect on the perceived appropriateness (b = .307, t(97) = 3.357, *p* = .001), in line with H3, and a positive effect on the perceived ability to learn (b = .283, t(97) = 2.963, *p* = .004). In turn, the perceived ability to learn had a positive effect on the perceived appropriateness (b = .351, t(96) = 3.844, *p* < .001). The indirect effect of the perceived anthropomorphism on the perceived appropriateness through the perceived ability to learn was also positive (b = .099, 95%CI[.026, .197]), in line with H4. The direct effect of the perceived anthropomorphism on the perceived appropriateness was positive (b = .208, t(96) = 2.328, p = .022). After dropping the demographic covariates (gender and age), the hypothesized effects remained significant (for the effect of perceived anthropomorphism on perceived ability to learn: t(99) = 2.855, p = .005; for the effect of perceived ability to learn on perceived appropriateness: t(98) = 4.276, p < .001; for the total effect of perceived anthropomorphism on perceived appropriateness: t(99) = 3.356, p = .001; for the indirect effect of perceived anthropomorphism on perceived appropriateness: 95% CI[.029, .209]). To rule out the possibility that perceived recommender anthropomorphism increased the perceived appropriateness of recommendations both for hedonic and utilitarian motives, a second-stage moderated mediation was run (Fig 5, PROCESS, Model 14, [78], 5000 bootstrap samples). The variables were the same as in the previous analysis, and the perceived recommendation hedonic (vs. utilitarian) motive was a second-stage moderator (VIFs < 1.4). The interaction effect of perceived ability to learn and perceived recommendation hedonic (vs. utilitarian) motive on the perceived appropriateness of hedonic appeals was positive (b = .096, t(94) = 2.074, p = .041). The conditional indirect effect of the perceived anthropomorphism on the perceived appropriateness at the perceived hedonic motive level of −1SD was non-significant (95% CI contained zero), while it was positive at the mean and +1SD perceived hedonic motive levels (B_mean_ = .064, 95%CI[.017, .126], and B_+1SD_ = .093, 95%CI[.026, .186]). That difference in the conditional effects was qualified by a positive moderated mediation index (B = .029) at 90% CI [.002, .078]. Noteworthily, the direct effect of perceived anthropomorphism on the perceived appropriateness turned non-significant in the moderated mediation model (p > .3), suggesting a full moderated mediation. After dropping the demographic covariates (gender and age), all the hypothesized effects remained significant (for the effect of perceived anthropomorphism on perceived ability to learn: t(99) = 2.855, p = .005; for the effect of perceived ability to learn on perceived appropriateness: t(96) = 4.853, p < .001; the interaction effect of perceived ability to learn and perceived recommendation hedonic (vs. utilitarian) motive on perceived appropriateness: t(96) = 2.264, p = .026), and the moderated moderation effect is even more visible (moderated mediation index: B = .031, 95% CI[.001, .092]). Those results indicate that the effect of the perceived anthropomorphism was indeed more positive for recommendations perceived as more hedonic (vs. utilitarian), strengthening the support for H3 and H4.

#### Discussion

Study 2 demonstrates the positive effect of the perceived online recommender anthropomorphism on the perceived appropriateness of its hedonic product appeals. Crucially, the results suggest the mediating role of the perceived online recommender ability to learn in the above relationship. The subtle anthropomorphic cues did not affect the perceived recommender anthropomorphism, contrary to Study 1, which applied the same cues. This suggests that subtle anthropomorphic cues may not work in some online contexts or consumer populations. However, Study 2 participants reacted to the recommender with different levels of perceived anthropomorphism, which appeared to affect their perceptions of the recommender’s ability to learn and the appropriateness of its hedonic appeals. This indicates that consumers differ in their tendency to anthropomorphize objects [80], like online recommenders, or how they react to specific ones. That variability may affect their reactions to the product recommendations.

## Conclusions

### Theoretical implications

The current research results extend the growing literature on consumer reaction to benefit-based and hedonic-based recommendations of AI-based systems [3–6] in two ways. First, the present studies involve the mediating role of the perceived online recommended ability to learn. Specifically, both in the case of benefit appeals and hedonic appeals, the current results suggest that perceiving an online recommender as more humanlike leads consumers to perceive it as more capable of understanding their needs and preferences, which in turn makes those consumers perceive the recommender more appropriate to present products through their benefits (vs. attributes) and hedonic (vs. utilitarian) purchase motives. Although the effects of perceived anthropomorphism and the ability to learn were studied in the context of AI-based recommenders’ benefit appeals [3, 4], the mediation relationship was not considered. Previous studies on consumer reactions to AI-based recommender hedonic appeals [5, 6] did not involve the perceived ability to learn. By evidencing a mediation of the relationship between perceived recommender anthropomorphism and the reaction to benefit and hedonic appeals through the perceived ability to learn, the present research shows the novel mechanism making consumers treat online recommender systems as humans.

Second, the dependent variable used in the current studies is the perceived appropriateness of product descriptions provided by online recommenders. Although Kim & Duhachek’s [4] research on consumer reactions to AI-based recommender benefit appeals involved perceived appropriateness, it was not linked to the perceived ability to learn. Perceived appropriateness was absent in the Wien and Peluso [6] research on consumer reactions to AI-based recommender hedonic appeals. Involving perceived appropriateness is theoretically meaningful because it supports applying schema congruency theory [13–16] to the context of online recommenders. Namely, the current results suggest that perceiving an online recommender as more humanlike, by making consumers perceive the recommender as more capable of understanding their needs and preferences, decreases the incongruity between the recommender’s output in the form of benefit and hedonic appeals and the typical "nonhumanness" schema associated with machines.

Noteworthily, the present research deals with online recommenders, while Kim & Duhachek [4] demonstrated the effect of anthropomorphism using a robot recommender. The difference between the recommender’s anthropomorphism in their study was related, among others, to describing the robot recommender as "conscious," which may not be relevant and realistic in the online context. In the current research (Study 1), the participants were exposed to both benefit-based and attribute-based recommendations (within-subject design), which may also be more realistic in the online context of choosing product alternatives in online shops compared to the between-subject design used by Kim & Duhachek [4].

### Practical implications

For marketers aiming to make AI-based online recommenders perceived more positively by consumers, the current results provide several suggestions. First, it is worth anthropomorphizing recommender systems, even using subtle cues like first-person messages, when communicating products through benefit language and hedonic purchase motives like fun and entertainment. If those cues are effective in increasing perceived system anthropomorphism in a given business context, as happened in Study 1, marketers can expect that benefit-based or hedonic-related recommendations of such recommender are more appreciated by users. Second, marketers can target benefit-based and hedonic-related recommendations at consumers tending to perceive system recommenders as humanlike. Namely, as suggested by the results of Study 2, anthropomorphic cues are not always effective, but people differ in their degree of perceived system anthropomorphism, which entails different responses to system recommendations. Highly-anthropomorphizing individuals may be identified, e.g., by online profiling in social media. The research found that an individual tendency to anthropomorphize is well predicted by cultural characteristics like collectivism [81] and personality traits like extroversion [82], both of which can manifest in a person’s social media activity. As those consumers are likely to perceive the system are more humanlike, they should value its benefit-based or hedonic-related recommendations more. Finally, marketers can attempt to convince consumers that an online recommender using benefit and hedonic appeals is capable of understanding the needs and preferences of its users by explaining to the recommender’s users how it can learn [4]. The mediating role of the perceived system ability to learn, evidenced in the current study, suggests that acting on that perception is more effective in improving consumer response to benefit-based and hedonic-related recommendations.

The corresponding conclusions may be relevant to consumer organizations and consumer protection authorities. Namely, consumers should be cautious about the benefit and hedonic product appeals provided by online recommenders, which are perceived as humanlike and able to learn. Consumers should be advised to consider whether the recommender is indeed competent enough to understand their perspective of using and enjoying the recommended products or just appears to be so.

### Limitations and further research directions

Five major limitations of the current studies call for further investigation. First, as the studies focused on the perceived appropriateness of benefit and hedonic appeals, the anthropomorphism and ability-to-learn effects on the persuasiveness of such appeals were not evidenced. Future research can, therefore, examine the entire causal path from perceived anthropomorphism to the appeals’ persuasiveness. Second, the subtle anthropomorphic cues of the fictitious recommender used in Study 2 were ineffective in altering the perceived recommender anthropomorphism. Further studies should try to overcome this, perhaps by strengthening the anthropomorphic cues, to better evidence the possibility of improving consumer reaction to hedonic appeals by anthropomorphizing the recommender. Third, the present studies relied on ostensible knowledge filtering based on gathering explicit personal information from users. At the same time, online recommender systems use other techniques like collaborative filtering based on the preferences of similar users. Suggesting that other people contributed to the machines’ recommendations may be another way to improve reactions to benefit and hedonic appeals, which is worth investigating. Fourth, as our dependent variable, perceived recommendation appropriateness, was measured using multiple objects (i.e., product descriptions), we used a single-item measurement to avoid participant fatigue. However, single-item measurement does allow for reliability assessment. Thus, further research can attempt to apply the multi-item measurement of perceived recommendation appropriateness, perhaps with a reduced set of product recommendations in stimuli. Lastly, the current studies were limited to smartphones as a product category and Polish young adults. The samples were homogenous and convenient, and women were overrepresented in the Study 2 sample. Thus, future studies need to cover other product categories and populations using representative consumer samples.

## Supporting information

S1 Table Study 1: Sample demographics across experimental conditions. (PDF)

S2 Table Study 1: SEM results for the serial mediation model testing H2. (PDF)

S3 Table Study 2: Sample demographics across experimental conditions. (PDF)

S1 Fig Study 1: The welcome page of the product recommendation website (across the experimental conditions). (PDF)

S2 Fig Study 2: The welcome page of the product recommendation website (across the experimental conditions). (PDF)
