# Supplementary material for: Online recommenders’ anthropomorphism improves user response to hedonic and benefit-based product appeals through the recommenders’ perceived ability to learn
Source: PLoS One. 2023 Jun 30;18(6):e0287663. doi: 10.1371/journal.pone.0287663 (PMC10313022; doi:10.1371/journal.pone.0287663)
Supplement: S1 Table — (PDF) [file pone.0287663.s001.pdf]

S1 Table

Study 1: Sample demographics across experimental conditions.

	Recommender anthropomorphism = HIGH		Recommender anthropomorphism = LOW	
	Frequency	Percent within condition	Frequency	Percent within condition
<u>Gender</u>				
**Females**	70	57.9	81	62.8
**Males**	51	42.1	48	37.2
<u>Age</u>				
**<25**	36	29.8	47	36.4
**≥25**	85	70.2	82	63.6
<u>Occupation</u>				
**Working**	93	76.9	89	69.0
**Studying**	30	24.8	35	27.1

	Website type = e-commerce		Website type = consumer organization	
	Frequency	Percent within condition	Frequency	Percent within condition
<u>Gender</u>				
**Females**	77	62.6	74	58.3
**Males**	46	37.4	53	41.7
<u>Age</u>				
**<25**	47	37.0	36	29.3
**≥25**	80	63.0	87	70.7
<u>Occupation</u>				
**Working**	89	72.4	93	73.2
**Studying**	26	20.2	39	32.2
