# Supplementary material for: Online recommenders’ anthropomorphism improves user response to hedonic and benefit-based product appeals through the recommenders’ perceived ability to learn
Source: PLoS One. 2023 Jun 30;18(6):e0287663. doi: 10.1371/journal.pone.0287663 (PMC10313022; doi:10.1371/journal.pone.0287663)
Supplement: S2 Table — (PDF) [file pone.0287663.s002.pdf]

S3 Table

Study 1: SEM results for the serial mediation model testing H2.

	**Standardized coefficient**	**z**	***p***
**Direct effects**			
recommender anthropomorphism condition (0=low, 1=high) → perceived recommender anthropomorphism	.226	3.72	< .001
perceived recommender anthropomorphism → perceived recommender ability to learn	.476	9.12	< .001
perceived recommender ability to learn → perceived appropriateness of benefit appeals	.407	6.13	< .001
recommender anthropomorphism condition (0=low, 1=high) → perceived recommender ability to learn	.183	3.22	< .001
perceived recommender anthropomorphism → perceived appropriateness of benefit appeals	.160	1.12	.264
recommender anthropomorphism condition (0=low, 1=high) → perceived appropriateness of benefit appeals	.064	1.13	.259
**Indirect effects on perceived appropriateness of benefit appeals**			
recommender anthropomorphism condition (0=low, 1=high)	.155	4.25	< .001
perceived recommender anthropomorphism	.194	1.86	.063
**Indirect effect on perceived recommender ability to learn**			
recommender anthropomorphism condition (0=low, 1=high)	.108	3.29	< .001
**Total effects on perceived appropriateness of benefit appeals**			
recommender anthropomorphism condition (0=low, 1=high)	.219	3.57	< .001
perceived recommender anthropomorphism	.354	3.40	< .001
**Total effect on perceived recommender ability to learn**			
recommender anthropomorphism condition (0=low, 1=high)	.291	4.54	< .001
