# Supplementary material for: Online recommenders’ anthropomorphism improves user response to hedonic and benefit-based product appeals through the recommenders’ perceived ability to learn
Source: PLoS One. 2023 Jun 30;18(6):e0287663. doi: 10.1371/journal.pone.0287663 (PMC10313022; doi:10.1371/journal.pone.0287663)
Supplement: S3 Table — (PDF) [file pone.0287663.s003.pdf]

S4 Table

Study 2: Sample demographics across experimental conditions.

	**Recommender anthropomorphism = HIGH**		**Recommender anthropomorphism = LOW**	
	**Frequency**	**Percent within condition**	**Frequency**	**Percent within condition**
**<u>Gender</u>**				
**Females**	45	86.5	42	84.0
**Males**	7	13.5	8	16.0
**<u>Age</u>**				
**<25**	34	65.4	26	52.0
**≥25**	18	34.6	24	48.0
**<u>Occupation</u>**				
**Working**	25	48.1	25	50.0
**Studying**	46	88.5	43	86.0
