# Supplementary material for: Online recommenders’ anthropomorphism improves user response to hedonic and benefit-based product appeals through the recommenders’ perceived ability to learn
Source: PLoS One. 2023 Jun 30;18(6):e0287663. doi: 10.1371/journal.pone.0287663 (PMC10313022; doi:10.1371/journal.pone.0287663)
Supplement: S1 Fig — (PDF) [file pone.0287663.s004.pdf]

S2 Fig

Study 1: The welcome page of the product recommendation website (across the experimental conditions).

Recommender anthropomorphism: HIGH

Website type: E-COMMERCE

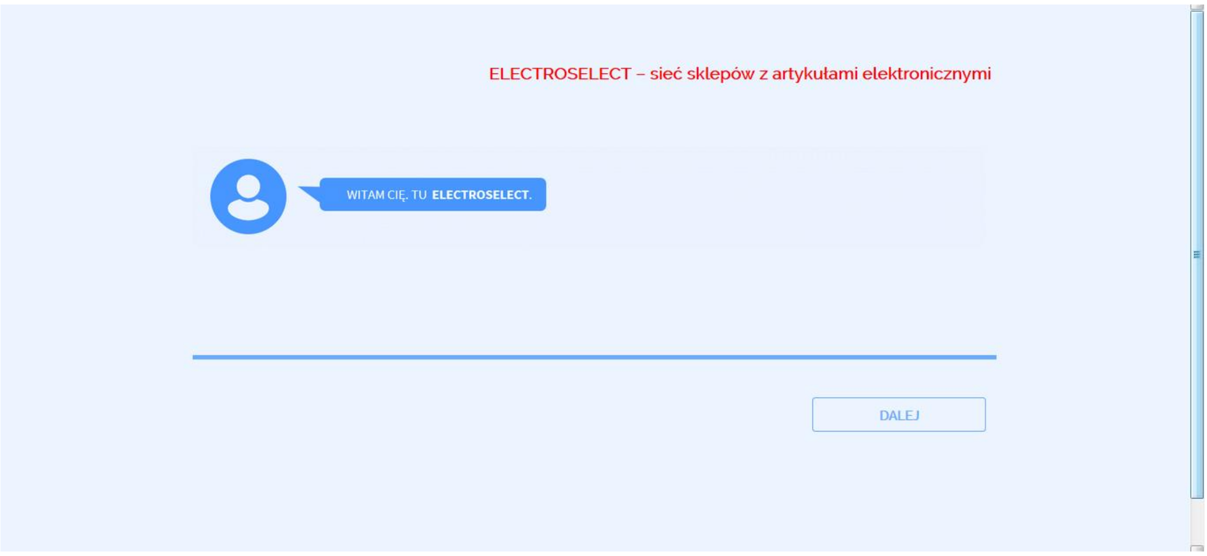

Translation:

ELECTROSELECT – a consumer electronics retailer

Hello, it is ELECTROSELECT here.

Recommender anthropomorphism: HIGH

Website type: CONSUMER ORGANIZATION

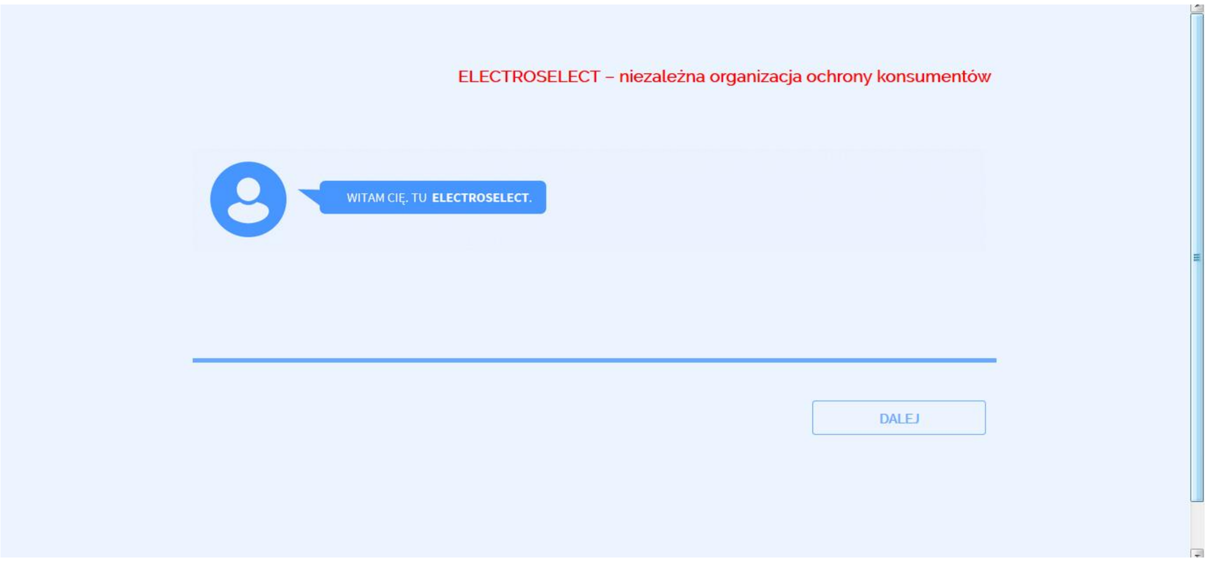

Translation:

ELECTROSELECT – a consumer electronics retailer

Hello, it is ELECTROSELECT here.

Recommender anthropomorphism: LOW

Website type: E-COMMERCE

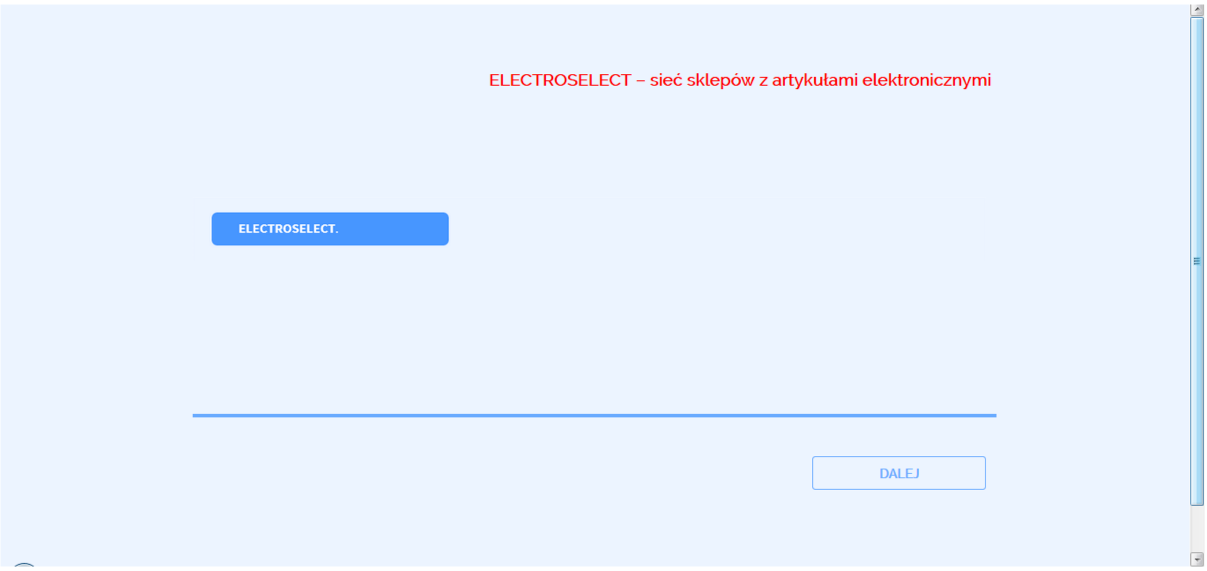

Translation:

ELECTROSELECT – a consumer electronics retailer

ELECTROSELECT.

Recommender anthropomorphism: LOW

Website type: CONSUMER ORGANIZATION

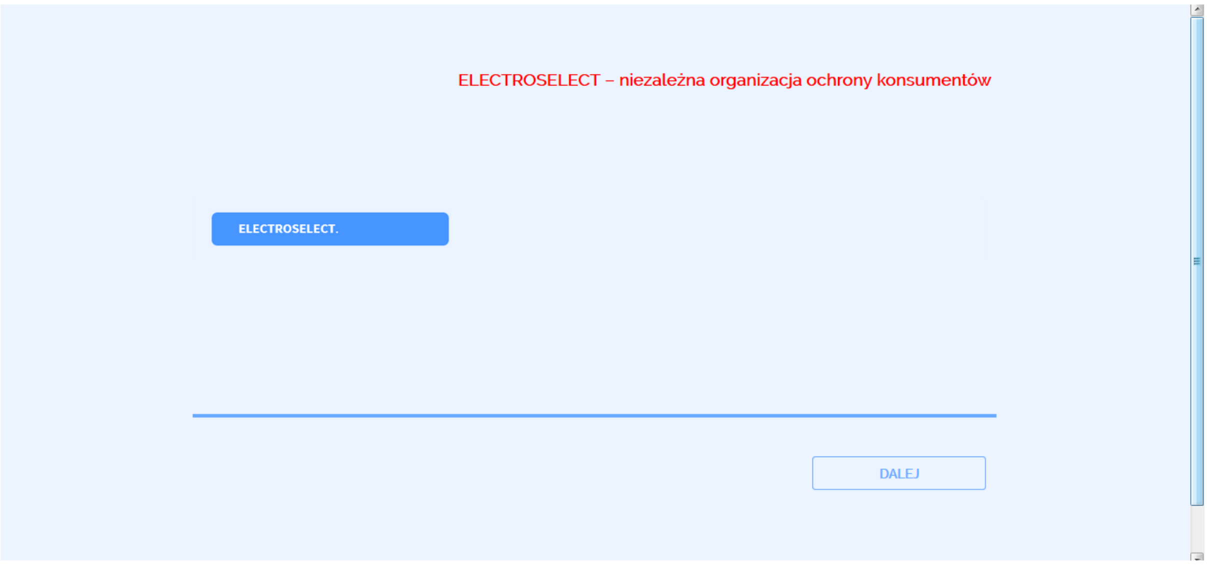

Translation:

ELECTROSELECT – an independent organization for consumer protection

ELECTROSELECT.
