# Supplementary material for: Online recommenders’ anthropomorphism improves user response to hedonic and benefit-based product appeals through the recommenders’ perceived ability to learn
Source: PLoS One. 2023 Jun 30;18(6):e0287663. doi: 10.1371/journal.pone.0287663 (PMC10313022; doi:10.1371/journal.pone.0287663)
Supplement: S2 Fig — (PDF) [file pone.0287663.s005.pdf]

S5 Fig

Study 2: The welcome page of the product recommendation website (across the experimental conditions).

Recommender anthropomorphism: HIGH

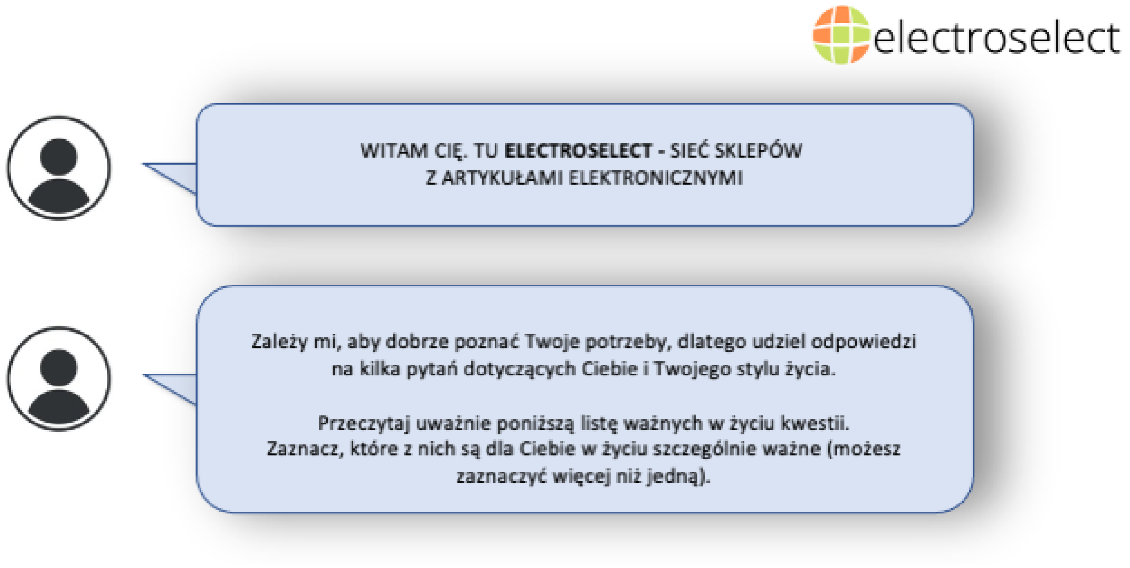

Translation:

Hello! It is ELECTROSELECT here - a consumer electronics retailer.

I want to learn about your needs. Therefore, respond on a few questions about you and your lifestyle.

Read carefully the below list of important life issues. Mark those especially important for you (you may mark more than one).

Recommender anthropomorphism: LOW

electroselect

ELECTROSELECT -SIEĆ SKLEPÓW Z ARTYKUŁAMI ELEKTRONICZNYMI

Aby można było dobrze poznać Twoje potrzeby, udziel odpowiedzi na kilka pytań dotyczących Ciebie i Twojego stylu życia.
Przeczytaj uważnie poniższą listę ważnych w życiu kwestii.
Zaznacz, które z nich są dla Ciebie w życiu szczególnie ważne (możesz zaznaczyć więcej niż jedną kwestię).

Translation:

ELECTROSELECT - a consumer electronics retailer.

To enable learning about your needs, respond on a few questions about you and your lifestyle.

Read carefully the below list of important life issues. Mark those especially important for you (you may mark more than one).
